# Cross-Ontology Multi-level Association Rule Mining in the Gene Ontology

**DOI:** 10.1371/journal.pone.0047411

**Published:** 2012-10-12

**Authors:** Prashanti Manda, Seval Ozkan, Hui Wang, Fiona McCarthy, Susan M. Bridges

**Affiliations:** 1 Department of Computer Science and Engineering, Mississippi State University, Mississippi State, Mississippi, United States of America; 2 Department of Plant and Soil Sciences, Mississippi State University, Mississippi State, Mississippi, United States of America; 3 Institute for Genomics, Biocomputing and Biotechnology, Mississippi State University, Mississippi State, Mississippi, United States of America; 4 Department of Basic Sciences, College of Veterinary Medicine, Mississippi State University, Mississippi State, Mississippi, United States of America; Saint Louis University, United States of America

## Abstract

The Gene Ontology (GO) has become the internationally accepted standard for representing function, process, and location aspects of gene products. The wealth of GO annotation data provides a valuable source of implicit knowledge of relationships among these aspects. We describe a new method for association rule mining to discover implicit co-occurrence relationships across the GO sub-ontologies at multiple levels of abstraction. Prior work on association rule mining in the GO has concentrated on mining knowledge at a single level of abstraction and/or between terms from the same sub-ontology. We have developed a bottom-up generalization procedure called Cross-Ontology Data Mining-Level by Level (COLL) that takes into account the structure and semantics of the GO, generates generalized transactions from annotation data and mines interesting multi-level cross-ontology association rules. We applied our method on publicly available chicken and mouse GO annotation datasets and mined 5368 and 3959 multi-level cross ontology rules from the two datasets respectively. We show that our approach discovers more and higher quality association rules from the GO as evaluated by biologists in comparison to previously published methods. Biologically interesting rules discovered by our method reveal unknown and surprising knowledge about co-occurring GO terms.

## Introduction

The Gene Ontology (GO) is the de facto standard for describing characteristics of gene products [1]. The rapid increase in the number of GO annotations from about 50 annotations in 1999 to more than 80 million by 2012 highlights the need for efficient data mining procedures for discovery of implicit knowledge in the annotation data [2], [3], [4], [5], [6]. We introduce an approach for mining interesting multi-level association rules across the three acyclic graphs used to represent the sub-ontologies of the GO: Cellular Component (CC), Molecular Function (MF) and Biological Process (BP).

Association Rule Mining (ARM) extracts implicit relationships between variables in a database *D  =  {t_1_, t_2_,…,t_m_}* [7]. The variables are represented as a set of binary attributes I * =  {i_1_, i_2_, i_3_*…*i_n_}* called items. A set of co-occurring items accompanied by an identifier is called a transaction. In the problem we are addressing, each transaction corresponds to a gene product and the attributes represent the presence or absence of a particular GO annotation. A rule is defined as an implication of the form 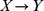 where 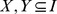 and 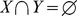 [_7_]. In our domain, the derived rules indicate implicit co-annotation patterns among a set of genes. Because we are mining cross-ontology rules, 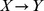
indicates that when GO term 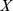
from one sub-ontology is associated with a set of genes in the dataset, GO term 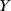
from a different sub-ontology is also likely to be associated with the same gene set.

Approaches for association rule mining can be broadly classified into single level ARM and multi-level ARM [8], [9], [10] depending on whether rules are mined from data at a single level of abstraction or at different levels of abstraction. Multi-level ARM requires that the data be represented using one or more ontologies in the form of hierarchies or directed acyclic graphs (DAG) such as the sub-ontologies of the GO. Terms near the top of the sub-ontologies are typically more abstract while those deep in the DAG are more specific. While the level of a term has been widely used as an indicator of its specificity, various studies have shown that all terms at the same level in the GO are not at the same specificity and information content level [11], [12]. Multi-level rule mining has the potential to overcome this issue by mining at multiple levels of the GO instead of focusing on a single level of detail. We show that the three sub-ontologies of the GO exhibit different distributions of terms across levels of abstraction in the structure of the GO and in annotations assigned to datasets. We have developed a bottom-up generalization procedure called Cross-Ontology Data Mining-Level by Level (COLL) for mining interesting multi-level association rules across the three sub-ontologies of the GO. COLL uses the structure and relationship semantics of the GO to translate data transactions into generalized/multi-level GO transactions before mining multi-level association rules. Monte Carlo simulation is used to determine the appropriate level for termination of generalization across sub-ontologies. An evaluation of the biological significance of the rules generated by COLL when applied to publicly available chicken and mouse GO annotation datasets demonstrates that our method produces more interesting rules compared to results from previously published ARM studies applied to the GO.

A number of research groups have used association rule mining to identify relationships among up and down regulated genes in gene expression studies [13], [14]. These studies do not make use of the GO and its hierarchical structure. Previous research applying association rule mining to the GO includes studies mining single level, multi-level and cross-ontology association rules [15], [16], [17], [18]. Carmona-Saez *et al.* [15] mine single level associations between GO annotations and expressed genes from microarray data integrated with GO annotation information. The approach does not utilize the inherent information provided by the GO structure thereby limiting the knowledge discovered.

In the area of cross-ontology association rule mining, other groups have developed methods for cross-ontology data mining to connect the three sub-ontologies of the GO with the goal of adding more biological information and more annotations. Burgun *et al.* [16] mine single level cross-ontology rules from publicly available GO annotation data. Myhre *et al.* [17] also mine single level cross-ontology rules connecting the three sub-ontologies and conduct an analysis of the discovered rules by biologists to demonstrate the utility of the rules. However, mining rules at a single conceptual level ignores information implied by the structure of the GO and limits the knowledge discovered.

In the area of multi-level association rule mining, Tseng *et al.* [18] discover multi-level association rules between GO terms annotated to up-regulated or down-regulated genes. Each transaction is the set of GO annotations associated with a gene. They achieve generalization by replacing each GO annotation with all of the GO terms on all of the paths from the term to the root of the ontology. This approach has two major shortcomings: 1) it will discover parent child relationships among terms that are already known, and 2) many of the rules will involve very high level GO terms with little information.

Other research had addressed generalization in the GO but for applications other than association rule mining. Davis *et al.* [19] describe an approach for generalizing in the GO by calculating the information content of a node using both the ontology structure and the annotation dataset as a metric for generalization. They use a non-traditional definition of information content of a concept *x* as *I_x_  =  P_x_−O_x_*, where *P_x_* is the information gained by not generalizing concept *x* and *O_x_* is the information lost if all the child terms of *x* are generalized to *x*. *P_x_* and *O_x_* are calculated using information from the annotation dataset and the ontology structure. They use this approach to generate automatic slim sets from the GO, but it is unclear how this approach will work for mining associations from multiple ontologies.

Hoehndorf et al. [20] describe a text-mining method for discovering significant associations between two DAGs and for conducting statistical testing of the significance of the discovered associations. The co-occurrence counts of pairs of vertices along with individual counts of the child vertices are used to assign scores to the vertex pairs. An association between two vertices is considered significant if the pair-wise score is high and the score decreases if one of the vertices is generalized or specialized any further indicating that the association is at the right level of abstraction. This method was used to identify cross-ontology associations across the GO and the Cell Ontology [21]. A disadvantage of this method is that it is computationally intensive since it generates all possible pairs between the vertices from the two DAGs and computes the scores between those pairs for multiple permutations before discovering the significant associations. The method has only been applied to text mining and not to mining annotation data.

In summary, prior efforts in association rule mining applied to annotation data from the GO focus on either mining multi-level association rules or cross-ontology rules, but not both. With more bio-ontologies being developed to describe different types of biological data and the increasing interest in using multiple ontologies to capture complex biological data, the ability to extract implicit relationships between different ontologies is becoming more important for biologists and tool developers who wish to utilize these ontologies and the data in them [22].

## Materials and Methods

### Generalization in the GO

Multi-level association rule mining requires viewing the GO annotation transactions at multiple levels of abstraction. We have chosen to use a generalization strategy for ontology traversal where the level of abstraction of the annotations is increased one level at a time with the Apriori algorithm [23] applied at each iteration. The termination level for generalization is determined using a Monte Carlo approach.

The cross-ontology data mining algorithm (COLL) presented below takes the following inputs:

1. A set of transactions T_Level_  =  {t_1_, t_2_ … t_m_} where each transaction t_i_ has a transaction identifier t_i,id_ accompanied by a list of terms: T_i_  =  t_i,id_, term_i,1_,term_i,2_…term_i,m_.

2. p: p-value threshold for the Chi-square test.

3. s: minimum support.

4. c: minimum confidence.

5. mf_cc_terminationlevel: Level of termination for cross-ontology categories MF → CC, CC → MF.

6. cc_bp_terminationlevel: Level of termination for cross-ontology categories CC → BP, BP → CC.

7. bp_mf_terminationlevel: Level of termination for cross-ontology categories BP → MF, MF → BP.

### Cross-Ontology Data Mining Level By Level (COLL)

#### Output

A set of non-redundant cross-ontology rules that satisfy the specified interestingness measure thresholds, R_Interesting  =  {R_1_, R_2_, R_3_…R_p_} where R_i_ contains a GO term as the antecedent and a GO term from a different sub-ontology as the consequent.

#### Functions

Apriori(p,s,c): Mines for association rules in the given transaction dataset.

FindParent(*term*): Finds parents of a given term in the hierarchy where the relation is *is-a* or *part-of*.

FindDeepestLevel(D): Finds the level of the deepest term in the provided dataset.

FindLevel(term): Finds the depth of any given term.

PruneSameOntology(R): Prunes all rules where the antecedent and consequent are from the same ontology.

FindCrossOntologyCategory(r): Returns the cross-ontology category of the rule.

#### Function COLL()

level ← FindDeepestLevel()

2 R_Interesting ← Φ

minlevel =  min(mf_ccterminationlevel,cc_bpterminationlevel,

bp_mfterminationlevel)

R ← *Apriori*( T_Level_, p,s,c)

R_Crossontology ← PruneSameOntology(R)

R_Interesting ← R_Interesting *U* R_Crossontology

Do while (level > minlevel)

For each t_i_ € T_Level_

For each term_i,j_ € t_i_

termlevel ← FindLevel(term_i,j_)

If termlevel  =  level

parentterm ← FindParent(term_i,j_)

 t_i_ ← t_i_ - { term_i,j_ } *U* {parentterm}

T_Level-1_ ← T_Level -1_ *U* t_i_

15 R ← *Apriori*( T_Level_, p)

16 R_Crossontology ← PruneSameOntology(R)

For each r_i_ € R_Crossontology

category  =  FindCrossOntologyCategory(r_i_)

If terminationlevel(category) < level

20 Rules_temp ← Rules_temp *U* r_i_

R_Interesting ← R_Interesting *U* Rules_temp

Rules_temp ← Φ

23 level ← level-1

The GO annotations in the transactions are typically at multiple levels in the GO hierarchy. Initially, T_Level_ is the original transaction set where *Level* represents the depth of the deepest annotation in the transaction set. The Apriori algorithm is applied to the initial set of transactions to generate a set of rules. All rules involving terms from the same ontology are pruned, and a set of interesting rules is established. Subsequently COLL replaces all GO annotations present at the current level with their immediate parent(s) related via an “*is-a” or* “*part-of”* relation to form a new transaction dataset, T_Level-1_. COLL then applies Apriori to the T_Level-1_ transactions, and adds new rules to the set of interesting rules. When both the antecedent and consequent GO terms come from the same ontology, they are removed, leaving only cross-ontology rules. These rules are classified into six categories depending on the GO sub-ontologies of the GO terms in the rule. COLL produces as output a set of non-redundant cross-ontology rules that satisfies the specified interestingness measure thresholds, R_Interesting  =  {R_1_, R_2_…R_p_ } where R_i_ contains a GO term as the antecedent and a GO term from a different sub-ontology as the consequent.

COLL terminates generalization based on individual termination levels for each category of cross-ontology rules. These termination levels are determined using synthetic datasets as described in the ‘Termination of Generalization’ section. COLL uses the highest termination level of the three cross-ontology categories to terminate the generalization and mining process. Rules from categories with lower termination levels are subsequently pruned. It should be noted that terms higher in the ontology have lower depth values.

**Figure 1:**
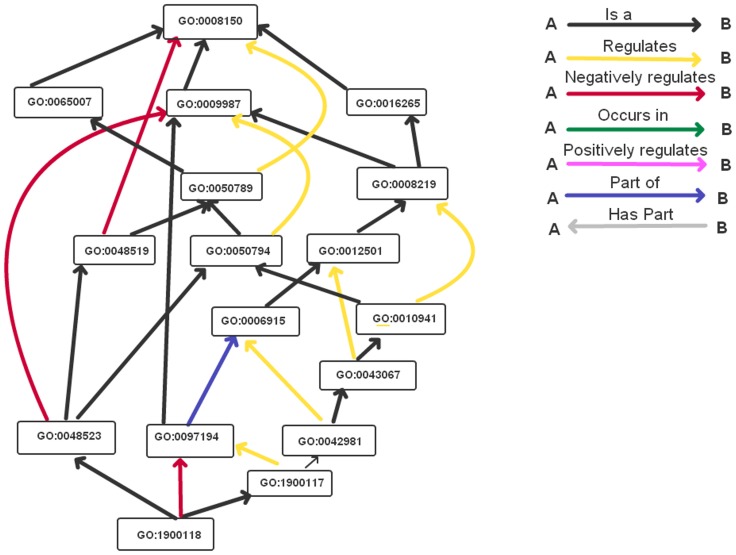
Issues in generalization in the Gene Ontology.

Figure 1 illustrates several issues that must be addressed when generalizing in the GO sub-ontologies. First, each term can have multiple parents and therefore the term must be replaced by all of its parents. This may result in multiple assignments of the same term to a gene. The union operator is used to avoid duplicates. The GO supports many different types of relationships [24] as illustrated in Figure 1 adapted from QuickGO [25]. Only “is-a” and “part-of” relationships are defined to be transitive and therefore generalization is limited to these relationships.

The GO ontology was parsed and loaded into relational database tables. COLL is implemented in Perl and uses mySQL to access GO data from the database. We use Christian Borgelt's implementation of the Apriori algorithm to mine association rules from the transactions at each level [26]. The code for COLL and information about other necessary components are deposited at Dryad: doi:10.5061/dryad.nr353. The user will require appropriate database tables with GO ontology data to execute COLL. The user supplies a p-value threshold for the Chi-square test and the Apriori algorithm prunes all rules with p-values that do not meet the threshold. COLL also prunes any rules where the antecedent and consequent are from the same sub-ontology of the GO.

### Termination of generalization

As COLL iteratively generalizes GO annotations in the transaction dataset one level at a time, the annotations in the rules become more abstract. Rules at very high levels of abstraction are less informative and more likely to have occurred by chance. We have developed and evaluated three Monte Carlo methods for determining the termination level for generalization. All three approaches generate synthetic random datasets, mine the random datasets for rules, and use this data to determine the false discovery rate for different levels of generalization. In the first approach, annotations are selected randomly from all three sub-ontologies in the GO using a uniform distribution (Uniform Random). In the second approach, selection of random annotations mirrors the distribution of GO annotations at each level in the target sub-ontology (Random By Ontology) while in the third approach GO annotations are sampled with replacement from the set of all three sub-ontologies (Sampling with Replacement). To test these approaches, we used as our target database the gene annotation dataset for chicken from AgBase, a website that provides gene annotations for animal and agricultural plant gene products [27]. The chicken dataset (downloaded as of 2/9/11) contains 6259 transactions. The mouse gene annotation dataset from AgBase (downloaded as of 12/12/11) used in additional experiments in subsequent sections of the paper contains 22880 transactions.

**Figure 2:**
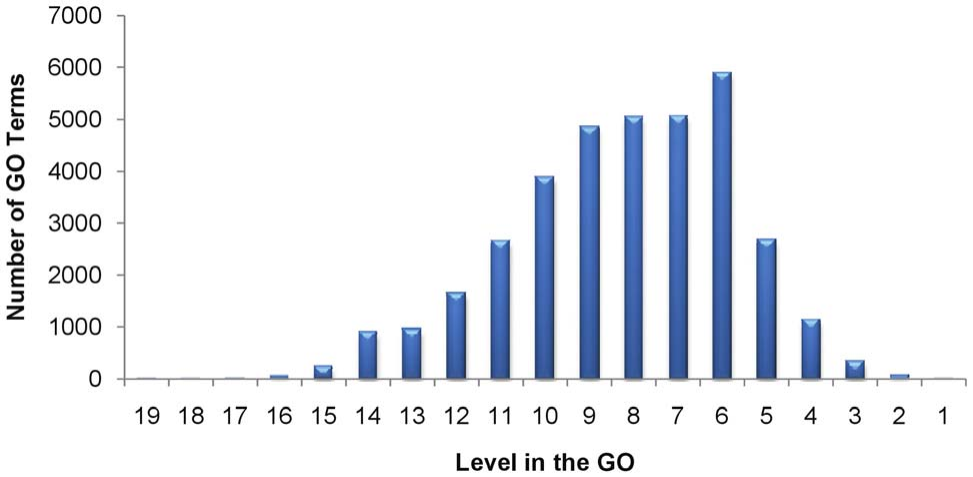
Number of terms at each level of the GO (data version 1.1.2633).

**Figure 3:**
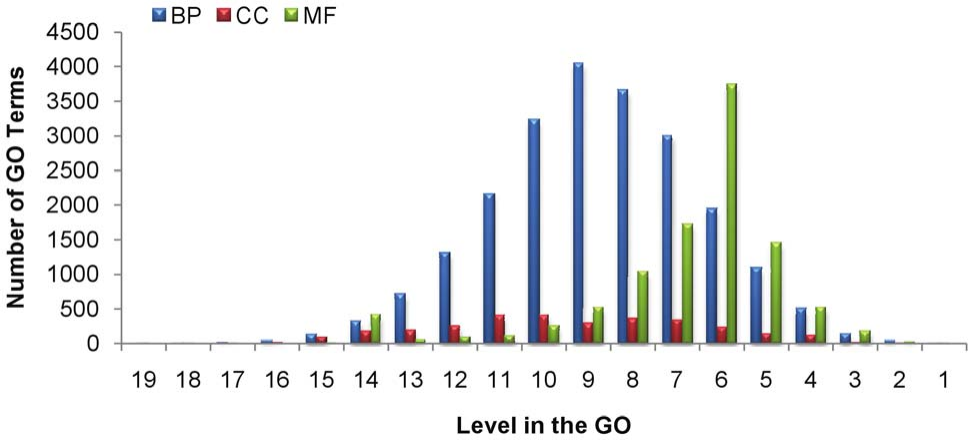
Distribution of terms in the GO (data version 1.1.2633) from different levels across CC, MF and BP.

The Uniform Random approach does not take into account the fact that terms in the GO are not distributed uniformly across different levels as shown in Figure 2. Additionally, the terms at any given level in the GO are not distributed uniformly across the sub-ontologies of the GO as shown in Figure 3.

The Random By Ontology approach models the GO annotation distribution in the target dataset to account for the uneven distribution of GO terms across different levels and sub-ontologies. A three step process is used to select each random GO annotation in the synthetic dataset. First, the distribution of GO annotations across the levels in the ontology is used to select the level of the GO term to be generated. Once a level has been selected, the distribution of annotations across sub-ontologies at the designated level is used to select a sub-ontology. Finally, an annotation is selected with uniform probability from the set of all GO terms at the designated level and sub-ontology.

The Sampling with Replacement approach uses all the GO annotations in the target dataset (including duplicates across transactions) as the background instead of all the GO terms in the GO. GO annotations are selected with a uniform probability with replacement from the background set.

The synthetic datasets are mined for multi-level cross-ontology rules in all six categories: MF → CC, CC → MF, CC → BP, BP → CC, BP → MF and MF → BP using algorithm COLL except that minlevel for generalization is set to 1. The false discovery rate (FDR) for each cross-ontology category at each generalization level is computed as FDR(CO_i_)  =  (CO_i_/R_i_) * 100, where *CO_i_* is the number of cross-ontology rules for cross-ontology category *CO* at generalization level *i* and *R_i_* is the total number of rules generated at generalization level _i_. The final false discovery rate for each cross-ontology category is the average FDR for 50 synthetic datasets. The termination level for each cross-ontology category is the first level of generalization where the FDR exceeds a predetermined threshold.

## Results And Discussion

The iterative generalization and mining method used by COLL explores many multi-level GO term combinations to discover implicit co-occurrence relationships. One of the limitations of this approach is that some multi-level term combinations get excluded because of the level-by-level generalization. We have explored a different method of generalization, which conducts inferences via transitive relationships in the GO such as “*is-a”* and “*part-of”* and supplements annotations with all inferred ancestors. This algorithm generalizes all annotations at the same time and then the generalized transactions are mined using the Apriori algorithm. A comparison of the results from the two methods revealed that the rules discovered by both approaches were very similar in terms of the quantity and the distribution across different levels of the GO. We chose the incremental generalization and mining approach since it discovers the more informative rules first.

### Termination Level

**Figure 4:**
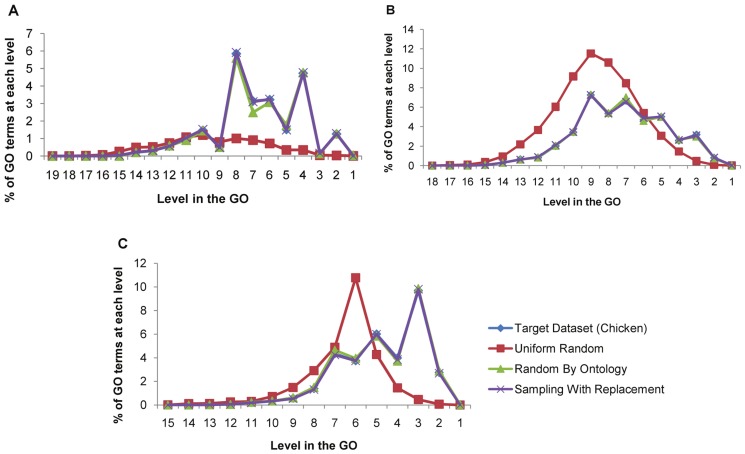
A comparison of the distribution of GO annotations in the synthetic datasets generated using the three approaches and the distribution in the target dataset in the three sub-ontologies: (a): Cellular Component, (b) Biological Process, (c) Molecular Function.

The results shown in Figure 4 show that both the Random By Ontology and Sampling with Replacement approaches generate synthetic datasets with GO distributions similar to the target dataset for all three sub-ontologies. The Uniform Random approach does not adequately model the distribution of GO terms in the target dataset. The Random By Ontology approach with an FDR threshold of 0.01 is used to determine termination levels in the remainder of the experiments.

Table 1 shows the FDR for each cross-ontology category at each level for the chicken dataset. Based on these results, the termination level for this dataset with an FDR of 0.01 is 6 for MF → CC, CC → MF, BP → MF, MF → BP and 8 for CC → BP, BP →CC.

**Table 1:** Average false discovery rate of random cross-ontology rules from 50 synthetic datasets at each level of generalization.

Level of Generalization in the GO	False Discovery Rate of Random Rules		
	MF → CC, CC →MF	BP → MF, MF → BP	CC → BP, BP →CC
16	0	0	0
15	0	0	0
14	0	0	0
13	0	0	0
12	0	0	0
11	0	0	0
10	0	0	0
9	0.00020	0.00032	0.00016
8	0.00150	0.00000	0.00422
7	0.00372	0.00032	0.01000
6	0.00438	0.00130	0.00924
5	0.02076	0.02088	0.01974
4	0.01724	0.03904	0.01644
3	0.01378	0.02792	0.04646

### Interestingness measures and pruning strategies

We use support, confidence and the Chi-square test as measures of interestingness during the rule mining process. A low support threshold and a high confidence threshold were used in the mining process. Unlike market basket applications where high support is required [7], [8], [9], [10], [23], [28], GO annotations that co-occur with a high frequency, even the terms each occur a relatively small number of times, are still interesting if they are not likely to occur together by chance. The support, s of a rule X → Y is calculated as the probability of X and Y co-occurring in the transaction dataset; s_X → Y_  =  P(X ∩ Y). The confidence, c of a rule X → Y is calculated as the probability of observing Y given that X is present in a transaction; c_X → Y_  =  P(Y|X). The Chi-square test compares the values of expected occurrence with the value of observed occurrence for every attribute in a transaction and reports a p-value which can be used to infer the level of dependence between two attributes [29], [30]. Previous research on mining multilevel association rules has used multiple support thresholds for different levels in the hierarchy but it can be very difficult to determine how these support thresholds should be calculated. The Chi-square test automatically addresses this issue by using the expected and observed occurrence counts for terms at different levels. The rules that pass the chi-square test threshold contain GO term pairs that occur more significantly than expected.

In addition to using interestingness measures to prune rules while mining, the following strategies are also used to prune rules that are biologically uninteresting:

1. Rules where the antecedent and the consequent are related by a child-ancestor relationship are pruned. Such relationships are implied by the true path rule in the GO and do not convey novel information to a biologist.
2. When the result set contains two rules of the form X → Y and X → Ancestor(Y) with a confidence difference of less than 10%, the rule of the form X → Ancestor(Y) is pruned. Given the rule X → Y, the rule X → Ancestor(Y) is implied and thus the more detailed version of the rule is retained.

### Association Rules

We applied the cross-ontology data mining algorithm to the chicken and mouse datasets with 0.05% support, 60% confidence and a p-value of 0.01 for the Chi-square test and compared these results with those resulting from applying a previously published approach described by Burgun *et al.* [16]. Burgun's approach does not use any generalization and thus, mines single level rules. Table 2 shows that, after pruning, COLL mines 5368 and 3959 cross-ontology rules from the chicken and mouse datasets respectively. Our pruning strategies reduce the total number of rules by 96.99% and 95.26% for the chicken and mouse datasets. The rules generated by Burgun *et al.* are a subset of the rules generated by COLL and do not include multi-level rules. COLL produced substantially more cross-ontology rules than Burgun's approach.

**Table 2:** Summary of the number of rules mined before and after pruning by COLL and the Burgun approach.

Dataset	COLL		BURGUN	
	Number of Rules Mined	Number of Cross-Ontology Rules after Pruning	Number of Rules Mined	Number of Cross-Ontology Rules after Pruning
Chicken	178362	5368	12422	2693
Mouse (All annotations)	83602	3959	4936	1517

It is to be noted that in this study, association rule mining discovers inherent patterns between GO annotations. These patterns are a result of co-annotation of one or more GO terms to a particular gene product. Therefore, the antecedent and consequent GO terms in our cross-ontology rules are existing GO terms from annotation data and not new terms.

COLL discovered rules at multiple levels of generalization from the chicken and mouse datasets in all six of the cross-ontology categories. Table 3 shows that the number of rules mined at each level of generalization increases from level 14 to level 6. This can be attributed to two facts. Firstly, generalization lends increased support to co-occurring GO term pairs thereby resulting in more rules. Secondly, the GO is more populated at levels 12 to 6, which results in the majority of generalization taking place at these levels thereby causing an increase in the mined rules. The number of rules from each cross-ontology category is shown in Table 4. The rules were categorized by their confidence values and the results in Table 5 show that a majority of the rules have a very high confidence level. Examples of the cross-ontology rules mined from the chicken dataset by COLL are shown in Table 6.

**Table 3:** Number of rules mined by COLL at each level of generalization mined from the chicken and mouse datasets.

Level of Generalization in the GO	Chicken All Annotations	Mouse	
		All Annotations	IEA AnnotationsRemoved
14	2	0	0
13	11	10	6
12	24	12	17
11	91	24	33
10	208	99	110
9	595	327	317
8	938	870	953
7	1467	1152	1562
6	2025	1465	2131

**Table 4:** Number of rules mined by COLL in each cross-ontology category.

Cross-Ontology Rule Category	Chicken All Annotations	Mouse	
		All Annotations	IEA AnnotationsRemoved
CC → BP	658	246	872
BP → CC	1669	1532	2129
MF → BP	1510	1240	1272
BP → MF	950	326	472
MF → CC	421	538	321
CC → MF	153	77	63

**Table 5:** Number of rules mined by COLL in each confidence range.

Cross-ontology Rule Category	Chicken All Annotations	Mouse	
		All Annotations	IEA AnnotationsRemoved
100%	1759	593	603
90%–99%	85	539	206
80%–89%	740	590	852
70%–79%	1196	792	942
60%–69%	1581	1445	2526

**Table 6:** Examples of cross-ontology rules mined from the chicken dataset.

Antecedent	GO Term Name	Consequent	GO Term Name	Cross-Ontology Category
GO:0005901	caveola	GO:0031325	positive regulation of cellular metabolic process	CC → BP
GO:0005929	cilium	GO:0042058	regulation of epidermal growth factor receptor signaling pathway	CC → BP
GO:0015491	cation:cation antiporter activity	GO:0045895	regulation of protein kinase activity	MF → BP
GO:0015491	cation:cation antiporter activity	GO:0015707	nitrite transport	MF → BP
GO:0043091	L-arginine import	GO:0051139	metal ion:hydrogen antiporter activity	BP → MF
GO:0002286	T cell activation involved in immune response	GO:0043231	intracellular membrane-bounded organelle	BP → CC
GO:0015491	cation:cation antiporter activity	GO:0045859	regulation of protein kinase activity	MF → BP
GO:0016459	myosin complex	GO:0003774	motor activity	CC → MF

In order to compare the biological relevance of the rules mined by the two approaches, two biologists manually evaluated rules selected from the two approaches. The biologists categorized rules into one of the three categories for surprisingness (Unknown/Surprising, Somewhat known and Widely known) and meaningfulness (Meaningful, Maybe meaningful and Not meaningful). The surprisingness of a rule determines if the relationship was hitherto unknown to the biologist. The meaningfulness of a rule indicates whether or not it makes sense for the items in the rule to be co-annotated. A brief description of these categories is as follows:

1. Surprisingness:
  - Unknown/Surprising: The rule reveals a relationship that the biologist had no prior knowledge of.
  - Somewhat known: There is limited knowledge on the relationship in the rule and might be useful for researchers.
  - Widely known: The relationship is an obvious one and is common knowledge.
2. Meaningfulness:
  - Meaningful: It seems acceptable to the biologist that the items in the rule were co-annotated.
  - Maybe meaningful: The items in the rule might be co-annotated in specific scenarios.
  - Not meaningful: The biologist does not see the reason behind co-annotating the items in the rule.

We conducted two evaluations with rule sets chosen using different selection strategies. For the first evaluation (Table 7), 25 rules were chosen at random from the mouse and chicken result sets and a biologist was asked to assign the rules to the categories shown in Table 7. In order to evaluate the effect of annotations inferred from electronic annotation (IEA) on rule surprisingness, the mouse dataset was also mined after removing all IEA annotations. Twenty-five random rules were evaluated from this list and the results are reported in Table 7.

**Table 7:** Number of rules in each evaluation category from a random set of 25 rules mined by COLL and the Burgun approach.

Evaluation Category		Number of Rules in Evaluation Category					
		ChickenAll Annotations		Mouse			
				All Annotations		IEA AnnotationsRemoved	
		COLL	Burgun	COLL	Burgun	COLL	Burgun
Surprisingness	Unknown/Surprising	5	0	4	1	0	1
	Somewhat Known	4	5	2	2	2	3
	Widely Known	15	18	19	22	18	17
Meaningfulness	Meaningful	16	22	19	22	19	19
	Maybe Meaningful	3	2	6	2	0	3
	Not Meaningful	5	0	0	0	0	0

For the second evaluation, we selected 50 rules with lower confidence values (60% to 64%) and 50 with the highest confidence values (100%) from the mouse dataset with all annotations. We noticed that the rules were largely dominated by rules involving Cellular Component (CC → BP, BP → CC, CC → MF, MF → CC). In order to ensure a good representation of rules from all categories, we selected 20 rules from CC → BP, BP → CC, CC → MF, MF → CC and 30 rules from MF → BP, BP → MF. All of the rules with 100% confidence derived by both methods were deemed to be widely known and meaningful by the biologists. These rules represent commonly known biological knowledge. The results for the evaluation of rules with lower confidence are reported in Table 8.

**Table 8:** Number of rules in each evaluation category from a set of 50 rules in a confidence range of 60–64% mined by COLL and the Burgun approach.

Evaluation Category		Mouse All Annotations	
		COLL	Burgun
Surprisingness	Unknown/Surprising	4	0
	Somewhat Known	8	3
	Widely Known	35	41
Meaningfulness	Meaningful	39	35
	Maybe Meaningful	11	11
	Not Meaningful	0	0

Both evaluations (Table 7, 8) show that COLL discovers unknown and surprising rules while none of the rules discovered by Burgun are surprising. The majority of rules identified by both approaches is biologically meaningful. However, most of the meaningful rules identified by Burgun are widely known and no surprising/unknown rules are discovered. In addition to discovering many more rules as compared to Burgun (49% more in chicken, 61% more in mouse) COLL discovers more unknown and surprising rules.

The evaluation of cross-ontology rules mined after all IEA annotations were removed revealed that no Unknown/Surprising rules are mined by the cross-ontology data mining algorithm for the selected subset. The biologists evaluated these rules based upon personal, biological knowledge and literature searches. In cases where there the GO annotation is based solely on literature, all GO annotations will be documented and found via literature searches. Since IEA derived GO annotations are based upon existing annotation knowledge (such as Enyzme Commission and SwissProt Keywords) and conserved functional motifs and domains (InterPro), the IEA annotations in effect represent derived biological knowledge that is applied generally rather than from a species-specific experiment.

## Conclusion

The Gene Ontology is a vast resource for understanding gene function and there are currently more than 80 million GO annotations available for a diverse range of species. Apart from containing gene product information, GO annotations contain a huge amount of implicit knowledge that can be discovered using data mining techniques such as association rule mining. In this study, we describe an approach for mining multi-level cross-ontology association rules from GO annotations using level-by-level generalization as the ontology traversal mechanism. The cross-ontology data mining algorithm views annotation data at varying levels of detail and captures implicit patterns of co-occurring GO terms across sub-ontologies. We show that COLL discovers more and better quality rules as compared to a previously published approach that mined single level cross-ontology rules.
